# Variation in photoperiod response corresponds to differences in circadian light sensitivity in northern and southern *Nasonia vitripennis* lines

**DOI:** 10.1007/s00359-023-01674-2

**Published:** 2023-10-18

**Authors:** Theresa S. E. Floessner, Elena Dalla Benetta, Domien G. M. Beersma, Roelof A. Hut

**Affiliations:** 1 Chronobiology Unit, Neurobiology Expertise Group, Groningen Institute for Evolutionary Life Sciences, University of Groningen, Groningen, the Netherlands; 2 Evolutionary Genetics, Development & Behaviour Expertise Group, Groningen Institute for Evolutionary Life Sciences, University of Groningen, Groningen, the Netherlands

**Keywords:** Photoperiodic response, Latitude, Circadian clock, External coincidence timer, Diapause, Hymenoptera

## Abstract

The circadian clock times physiological and behavioural processes and resets on a daily basis to synchronize with the environment. The involvement of the circadian clock in photoperiodic time measurement synchronising annual rhythms is still under debate and different models have been proposed explaining their integration. Insects overcome unfavourable conditions in diapause, a form of dormancy. A latitudinal cline in diapause induction in the parasitoid wasp *Nasonia vitripennis* as well as a difference in circadian light sensitivity between north and south provide us with additional evidence that the circadian system of *Nasonia* is involved in photoperiodic time measurement and that latitude-specific seasonality drives adaptive evolution in photoperiodism partly through adaptation responses in the circadian system. We tested diapause induction in a range of T-cycles and photoperiods and found diapause induction in short photoperiods in all T-cycles in the northern line but in the southern line, diapause only occurred in T-cycles close to 24 h. Due to a lower light sensitivity in the southern line, a wider distribution of phase angles of entrainment can be expected at a specific T-cycle duration, while the range of entrainment will decrease. Taking these oscillator properties into account, our data can be explained by an external coincidence model involving a single oscillator with a light-sensitive phase that drives annual timing of diapause in *Nasonia vitripennis*.

**Supplementary Information:**

The online version contains supplementary material available at 10.1007/s00359-023-01674-2.

## Introduction

### Seasonality, photoperiodism and the circadian system

Organisms exist in a dynamic environment, with periodically occurring daily and seasonal changes. It is necessary for survival to initiate physiological processes in anticipation of upcoming seasonal changes. Therefore, photoperiodic time measurements evolved, which determine the time of year by measuring day length (Danilevskii 1965 Chapter 2, Hut et al. 2013). In insects, seasonal changes have led to adaptive temporal organisation of seasonal growth, reproduction and diapause (dormancy). Responses to seasonal changes in day length drive seasonal physiology (Marcovitch 1923). This requires a neurophysiological system that measures either night length (scotoperiod), day length (photoperiod) or both. The sensitive period in which an organism will respond to this photoperiodic stimulus either occurs in the life cycles of the organism itself (inducing adult diapause as in *Drosophila melanogaster* Saunders et al. 1989) or in the parental generation which leads to diapause in egg or larval state (larval diapause in *Nasonia vitripennis* Saunders 1965a, 1965b*)*. It has been studied intensively whether the night or the day length is the crucial component by inducing diapause (see review by Saunders (2013)) and it seems that in many tested insect species the duration of darkness is the determining signal (Saunders 1973), although the duration of the light phase as critical signal has also been found (Saunders 1987). In *Nasonia*, as in some other insect species, seasonal timing involves the circadian system (Saunders 1968, 1974, 1990), but in other species, proof of circadian involvement seems absent (Pittendrigh et al. 1970; Pittendrigh and Minis 1971; Lees 1990). In mammals, a seasonal timing mechanism requires the involvement of the circadian system and circadian clock genes (Dardente et al. 2010; Masumoto et al. 2010). Although light is the main inductive stimulus, also temperature, food availability, population density or humidity can influence seasonal response (Saunders 1966a, b, 1970; Tauber et al. 1986; Hodkova and Socha 1995; Christiansen-Weniger and Hardie 1999; Saunders et al. 2002 Chapter 10; Danilevskii 1965 Chapter 3,4,5).

A model explaining photoperiodic time measurement and the involvement of a circadian clock was firstly proposed by Bünning (1936). He proposed a dual-phasic oscillator with a frequency of approximately 24 h, consisting of a photophil phase of 12 h that required light and a scotophil phase of also 12 h requiring darkness. A short day response, for example, diapause is expected when light only occurs during photophil, whereas a long day response will occur when the light is extended into scotophil phase. Thus, Bünning combined the daily time measurement of the circadian clock with the measurement of the darkness duration to form an internal representation of day length required for the seasonal response. An alternative system not involving a self-sustainable oscillator is the hourglass model (Lees 1973). It requires a daily stimulation of the system by an external light–dark cycle. The system measures either the length of the light or dark phase by a homeostatic process which is reset every day, and crosses a threshold after a critical duration of the light or dark phase. Bünning’s original oscillator theory was taken further by Pittendrigh (Pittendrigh and Minis 1964; Pittendrigh 1966) by describing an external coincidence model which states the occurrence of short day response (e.g. diapause) when a single oscillator’s light-sensitive phase coincides with environmental darkness and a long day response when the light-sensitive phase coincides with light. In the internal coincidence model, Pittendrigh (1972) proposed two oscillators: one coupled to dawn and one to dusk. Their phase angle difference forms an internal representation of day length and determines seasonal status of the organism. In addition, the internal and external coincidence models, describing the involvement of the circadian system in photoperiodism, may lead to different predictions for latitudinal selection pressure on circadian properties like free-running period and light sensitivity (Hut and Beersma 2011).

### Critical photoperiod and latitudinal cline

Insects can survive unfavourable conditions by going into diapause. Depending on the species, diapause can occur at different stages of the life cycle (Tauber et al. 1986) and usually occurs when photoperiod decreases to a specific threshold: the critical photoperiod (CPP). Seasonal variation in temperature and day length increases with distance to the equator, while the average environmental temperature decreases (Hut et al. 2013). Together, this led to the hypothesis that CPP should increase with latitude to form a so-called latitudinal cline. Data sets from many insect species confirmed a latitudinal cline in CPP; insect populations from higher latitudes show diapause at longer photoperiods than their relatives from lower latitudes (Danilevskii 1965 Chapter 5; Hut et al. 2013). The latitudinal differences in photoperiodic response and critical daylength was shown to have a genetic origin in the pitcher plant mosquito (Mathias et al. 2006). Paolucci et al. (2013) showed in an extensive study a latitudinal cline in CPP, where northern *Nasonia vitripennis* populations have longer CPP than the southern populations. *Nasonia* shows a distinct short photoperiod maternal response which induces diapause in the offspring larva (Saunders 1965a, b, 1966a, b). In addition, *Nasonia* adults expresses a strong and stable circadian activity rhythm with a clear diurnal pattern (Bartossa et al. 2013; Floessner et al. 2019). These characteristics and its global occurrence are features that make *Nasonia* an appropriate study species for seasonal and circadian environmental adaptation. Furthermore, quantitative trait locus (QTL) analysis discovered two genomic regions involved in diapause induction (Paolucci et al. 2016). Among other genes, these regions (on chromosome 1 and 5) encode for three important clock genes *period*, *cycle* and *cryptochrome*. Further analysis of the *period* gene showed latitudinal variation of mainly two haplotypes, one with higher frequency in northern populations and the other with higher frequency in southern populations (Paolucci et al. 2016). Additionally, we have determined longer circadian free-running periods in a northern *Nasonia* line compared to a southern line (Floessner et al. 2019).

Latitudinal clines are seen in many biological aspects and are usually considered to be an adaptive evolutionary response to a latitudinal environmental gradient (Hut et al. 2013). They can be used to trace evolutionary selection pressures for specific processes and may, therefore, be informative to find essential elements (genes) in physiological pathways of interest.

### General aim

Here, we aim to extend our knowledge about the involvement of the circadian system in photoperiodic response to the mechanism that drives latitudinal adaptation of *Nasonia vitripennis* by exposing a northern and a southern line to a partial Nanda-Hamner protocol. The full Nanda-Hamner protocol uses a wide combination array of photoperiods and cycle durations (T-cycles; Nanda and Hamner 1958). Briefly, when a photoperiodic response occurs under 24 h cycles (or multiples thereof), but not under non-24 h T-cycles, an involvement of the circadian system in photoperiodic response is concluded. Those “positive” responses were measured for example in *Nasonia vitripennis* (Saunders 1968, 1974) and *Drosophila melanogaster* (Saunders 1990). But also “negative” results have been reported in the Pink bollworm *Pectinophora gossypiella* (Pittendrigh et al. 1970; Pittendrigh and Minis 1971) or the pea aphid *Acyrthosiphon pisum* (Lees 1990) showing a non-circadian response or an hourglass timing system (Saunders 1974). The full Nanda-Hamner protocol in *Nasonia* provided evidence for the involvement of a circadian internal coincidence timing system (Saunders 1974). This finding allowed us to define a partial Nanda-Hamner protocol with a limited range of T-cycles around 24 h, aimed to distinguish differences between a northern and southern line of *Nasonia vitripennis,* in which a latitudinal cline in critical photoperiod and in circadian period have been described previously (Paolucci et al. 2013). If the circadian system is involved in photoperiodism, then possible differences in circadian light sensitivity between these lines could potentially result in observable differences in diapause responses to a partial Nanda-Hamner protocol, because circadian light sensitivity can affect the phase angle of circadian entrainment and thus the location of the time window for the photo inducible phase to break diapause. Together, this would provide additional evidence for circadian involvement in photoperiodism in *Nasonia vitripennis*. To strengthen our interpretation, it would help to include our results to a third intermediate population. We therefore compare our results to those presented by Saunders (1974), who worked with another *Nasonia* line, collected at an intermediate latitude.

## Materials and methods

### Experimental lines

Experiments were performed with two *Nasonia vitripennis* isofemale lines that had been established from single females originating from Oulu, Finland (northern line; 65°3′40.16’’N, 25°31′40.80’’E) and Corsica, France (southern line; 42°22′40.80’’N, 8°44′52.80’’E; Paolucci et al. 2013). Both lines were reared in temperature and humidity controlled climate chambers at 20° C (± 1 °C), 50–55% RH, and a light–dark cycle of 16 h light: 8 h dark (LD16:8). The wasps used during the experiment were F1 generations of individually housed females, supplied with two *Calliphora spp*. pupae as hosts.

### Entrainment period and test period

**Fig. 1:**
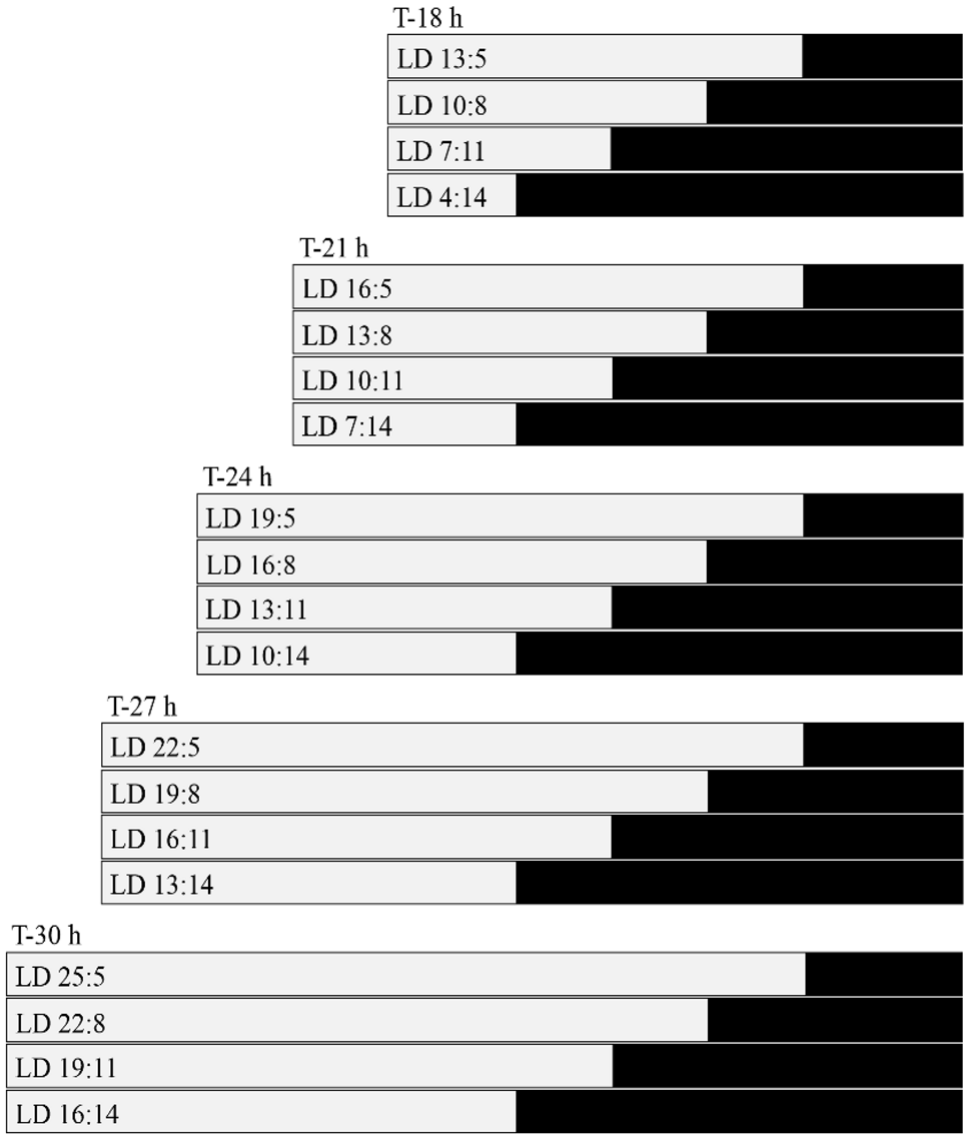
Light–dark regimes of the entrainment and test phase (day 1–18); five different T-cycles and four different photoperiods per T-cycle. For each T-cycle, we used photoperiods with dark periods of 5 h, 8 h, 11 h and 14 h. Bars represent T-cycle length, with the light phase (grey) and dark phase (black)

We used a partial Nanda-Hamner protocol to distinguish between photoperiodic responses in northern and southern lines. The experimental set up follows closely Saunders protocol that he applied during the T-cycle measurements of *Nasonia vitripennis* (Saunders 1968, 1974) to enable reliable comparisons. The light regime was designed to distinguish night length driven responses from day length driven responses (Vaze and Helfrich-Förster 2016). In our partial Nanda-Hamner protocol, groups of wasps were placed under various light–dark conditions using 5 different light–dark cycle durations (or T-cycles, of T-18 h, T-21 h, T-24 h, T-27 h, T-30 h) and within each T-cycle four different night lengths of 5, 8, 11 and 14 h and a variable number of hours in light adding up to the required T-cycle (Fig. 1). The light regime for each group was maintained throughout the entire experiment and did not change. During the entrainment period (day 1 to day 16) and test period (day 16 to day 18), the wasps were exposed to the experimental light–dark regimes (Fig. 1). For each experimental group, we prepared three mass culture vials per line containing ~ 30 females, ~ 5 males and ~ 30 hosts. For each LD cycle group, there were 33 parasitized pupae on average (range 22–64). On day 8, fresh host pupae were added during the light phase to feed the wasps.

For the test period (day 16 to day 18) individual females, 25 per experimental group, were transferred into acrylic test tubes and supplied with 2 fresh *Calliphora spp.* pupae as hosts. Females were removed 48 h later (in the end of the light phase of day 18) and the test tubes transferred into constant darkness for 3 more weeks so the eggs could develop into diapausing larvae or develop into pupae. The experiment was performed in light-tight boxes (23 × 14 × 32 cm) at 18 °C (± 1 °C). Each light-tight box was illuminated with one LED light source (Neutral White 4000 K PowerStar, Berkshire) of 2.1 · 10^15^ photons·cm^−2^·s^−1^.

### Diapause assessment

To score diapause response, the two host pupae in each test tube were opened and the status of the offspring was assessed. Diapausing offspring was hold in a larval state whereas developing offspring had developed into adults in the meantime of 3 weeks. A host pupa was counted as ‘diapausing’ when ≥ 50% of the progeny was in diapause, in the vast majority the offspring was either entirely diapausing or entirely developing (non-diapausing). Host pupae that were not parasitized were excluded from further calculations. For each light cycle, the results were calculated as the percentage of host pupae containing diapausing offspring during the test period.

### Circadian light sensitivity essay

To establish a full profile of circadian light sensitivity, we quantified circadian phase shifting response to five different white light pulse durations (0.3, 1, 4, 8, 16 h) and three different light intensities (9.37*10^13^ (low); 2.62 · 10^14^ (intermediate); 2.10 · 10^15^ (high) photons·cm-2·s-1) at 1-h and 4-h light pulse duration in an Aschoff type II protocol. In this protocol, newly eclosed wasps were entrained for 4 days to a 16:8 h LD cycle before released in continuous darkness. After 2 days in DD, a light pulse was provided at different time points across the circadian cycle (Suppl.Info). The circadian phase shifts calculated relative to a dark control and plotted as phase response curves (PRCs) for males and females of the northern and southern strain, resulting in a set of 44 PRC’s, which were classified as type 1 (weak resetting) or type 0 (strong resetting) by viewing the data as phase transition curves. Integration over a portion of the phase response curve (between ZT0 and ZT16) provided a single value for each PRC that represents the total phase shifting capacity for that specific photon dose over a section of the PRC that is most relevant for entrainment. All 44 phase shift capacity values were subsequently plotted against the total photon dose of the stimulation, combining variation in pulse duration and light intensity treatments. The resulting analysis provides a complete data set to establish differences in circadian light sensitivity for males and females of the different strains. See Supplementary Information for detailed methods.

## Results

**Fig. 2:**
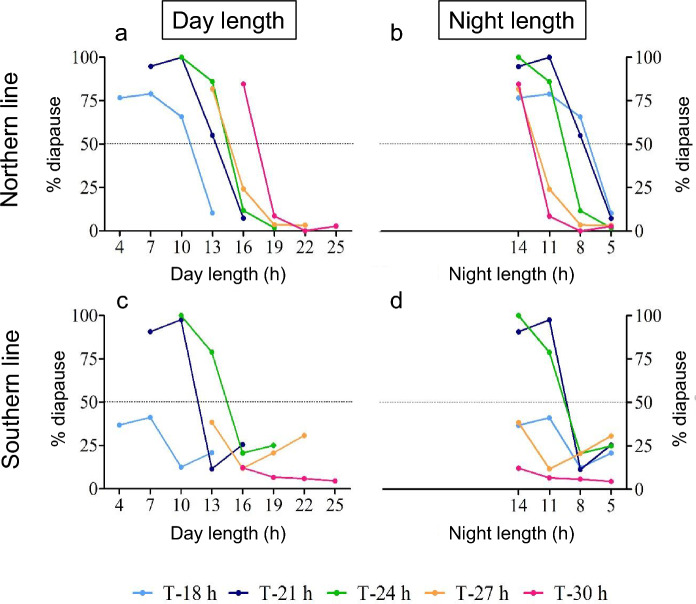
Photoperiodic response curve for diapause induction of Nasonia vitripennis lines from a **a**, **b** northern and a southern **c**, **d** European origin in different light regimes of different T-cycles and photoperiods. Diapause incidence is plotted as percentage diapause as a function of day length (**a**, **c**) or as a function of night length (**b**, **d**)

Both lines showed transitions from non-diapausing offspring to diapausing offspring (Fig. 2). Comparing diapause incidence within lines and between the different T-cycles, we noticed in the northern line that for all T-cycles a steep transition from non-diapausing offspring to diapausing offspring occurred at longer photoperiods (Fig. 2a, b), although 100% diapause was not reached in T-18 h, T-27 h and T-30 h (Fig. 2a, b). In T-18 h, the critical photoperiod was the shortest of all T-cycles, followed by T-21 h. T-24 h and T-27 h showed a nearly identical CPP and in T-30 h the CPP was the longest. In summary, the northern line showed diapause response at all T-cycles.

In the southern line, diapausing offspring was only observed in T-21 h and T-24 h. T-21 h expressed a shorter CPP than T-24 h (Fig. 2c, d). The other T-cycles resulted for all LD regimes in less than 50% diapause. In T-30 h, we measured 4–12% diapause incidence for all LD cycles (Fig. 2c, d).

Comparing both lines with each other, we see that the northern line responded stronger and with distinct response to the different T-cycle regimes than the southern line. The southern line did not show diapause response when T-cycles deviation from 24 h was large, especially when T was longer than 24 h.

Vaze and Helfrich-Förster (2016) raised the question whether the absolute day length or rather the night length was measured by the photoperiodic system. Therefore, we replotted our data also as a function of absolute night length expecting that when absolute night length is measured, the curves would superimpose. A logistic regression model was used within each strain and day length or night length analysis to determine significant differences in inflection points between T-cycles, using identical slopes. For the northern strain, the analyses showed a highly significant dependency of diapause on day length, when different constants were estimated for each T-cycle treatment (Fig. 2a, b; *n* = 24, df = 18, dev. = 103.79, *p* < 0.0001). For the southern strain, the analyses showed a highly significant dependency of diapause on day length, when different constants were estimated for each T-cycle treatment (Fig. 2c, d; *n* = 24, df = 18, dev. = 113.28, *p* < 0.0001). The variation in inflection points for each T-cycle did not differ within each strain between the day length and night length analysis (Northern strain: sd_day_ = 2.82, sd_night_ = 2.24; F_4,4_ = 1.59, *p* > 0.33; Southern strain: sd_day_ = 4.05, sd_night_ = 4.21; F_4,4_ = 1.08, *p* > 0.47). Thus, diapause incidence plotted against night length (Fig. 2b, d) does not show better superposition than when diapause is plotted against day length (Fig. 2a, c), suggesting that both day length and night length influence diapause induction in both *Nasonia* strains.

**Fig. 3:**
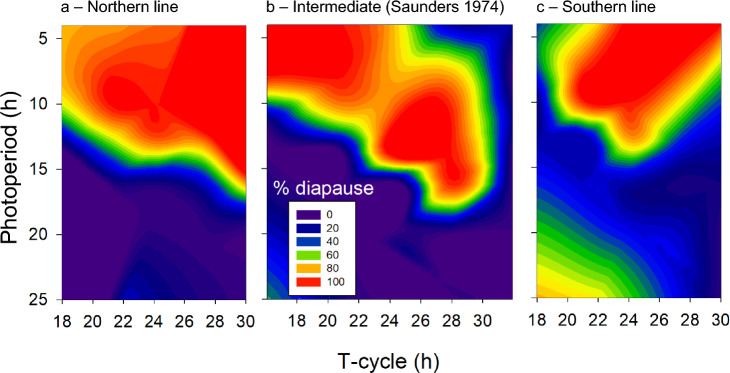
Circadian photoperiod landscapes for diapause response—comparison with Saunders 1974. Diapause response data for the northern (**a**) and southern (**c**) line are replotted as contour landscapes against T-cycle duration and photoperiod and compared with Saunders’ 1974 data (**b**), which were obtained from a *Nasonia vitripennis* line that originated from an intermediate latitude (Cambridge, UK; only data were plotted from similar T-cycle and photoperiod ranges as we used in Fig. 2)

In his complete Nanda-Hamner study, David Saunders used a *Nasonia* line originating from Cambridge, United Kingdom (52°12′19.213’’N, 0°7′18.541’’E; Saunders 1968, 1974), an intermediate latitudinal location between Oulu, Finland (65°3′40.16’’N, 25°31′40.80’’E) and Corsica, France (42°22′40.80’’N, 8°44′52.80’’E). Re-plotting Saunders results (Fig. 3) from T-cycles similar to cycle lengths that we used, we find similarities to both our northern and the southern data (Figs. 2a, c & 3). T-16 h, T-21 h, T-24 h, T-28 h show diapause induction at short photoperiods. T-32 h does not show more than 20% diapause induction, similar to results shown by the southern line (Fig. 2c). We conclude that the results of Saunders also support our results since those data show intermediate diapause induction responses when compared to our northern and southern data, while the geographical origin of the Saunders strain was also intermediate to the strains we used.

To enable interpretation of the differences in photoperiodic responses between the northern and southern line, we investigated whether both lines may differ in their circadian light sensitivity. Five different white light pulse durations (0.3, 1, 4, 8, 16 h; Fig. s1, s2) and three different light intensities (9.37*10^13^ (low); 2.62 · 10^14^ (intermediate); 2.10 · 10^15^ (high) photons·cm-2·s-1) at 1-h (Figs. s3, s4) and 4-h (Figs. s5, s6) light pulse duration in an Aschoff type II protocol. Light pulses were provided at 12 time points spread out over a 24-h time axis and circadian phase shifts were quantified to provide 44 PRCs for males and females of the northern and southern strain. The resulting PRCs were also plotted as phase transition curves to enable classification as weak (type 1) or strong (type 2) circadian phase resetting. With increasing light pulse duration, males and females of the Northern line consentingly switch to strong resetting at lower stimulus strength than the southern line, indicating that the circadian system of the northern line is more sensitive to light (Table 1).

**Table 1:** Circadian phase resetting to different light pulse durations (0.3-h, 1-h, 4-h, 8-h, 16-h) of high light intensity in females and males from the northern and southern line

Pulse duration (h)	Female		Male	
	North	South	North	South
0.3	1	1	1	1
1	1	1	0	1
4	0	1	0	0
8	0	0	0	0
16	0	0	0	0

Responses became stronger (type 0) with longer light pulse durations. The transition from weak (type 1) to strong response occurs at shorter stimulus durations in the northern line than in the southern line. In both lines, males transitioned to strong resetting at shorter pulse durations than females

Likewise, at different light intensities, we see that males from only the northern line show strong circadian resetting to a 1-h light stimulus, while females from only the northern line show strong resetting to a 4-h light stimulus. This also indicates higher circadian light sensitivity in the northern line (Table 2).

**Table 2:** Circadian phase resetting to different light intensities at 1-h and 4-h pulses, for males and females from the northern and southern line

	1-h light pulse				4-h light pulse			
	Female		Male		Female		Male	
Light intensity	North	South	North	South	North	South	North	South
Low	1	1	0	1	0	1	0	0
Intermediate	1	1	0	1	0	1	0	0
High	1	1	0	1	0	1	0	0

Main differences are found between light pulse durations where northern females and southern males increased their responses from weak to strong when light pulse duration increased. Light intensity range was not large enough to elicit differences in response type within each group

**Fig. 4:**
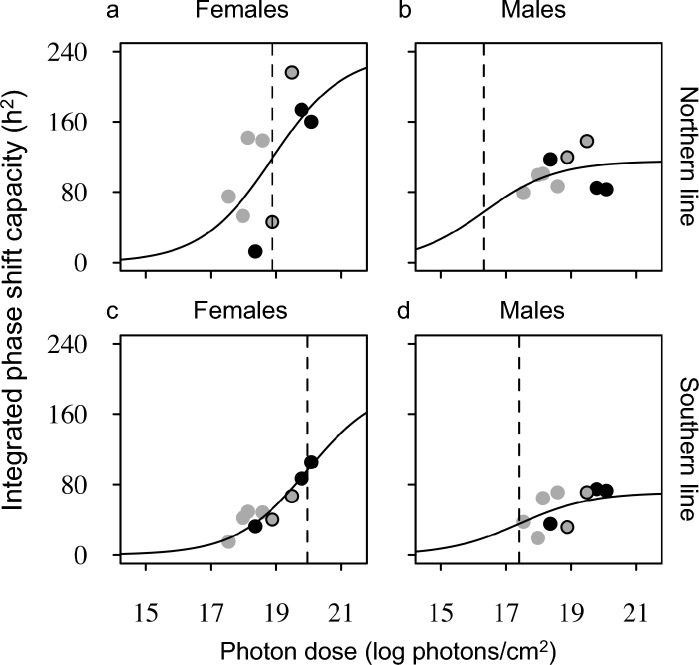
Photon dose response curves for females and males from northern and southern lines. Each point is the integration of a phase response curve (area-under-curve over ZT0-16). Integrated values originate from light pulse duration experiments at high light intensity (Figs. s1 & s2, grey), light pulse intensity experiments using 1 h or 4 h light pulses (Figs. s3– s6, black), or both (grey filled black circle). Dashed lines mark the curve inflection points, indicating higher response magnitude, but lower circadian light sensitivity in females (**a**, **c**) than in males (**b**, **d**), and higher circadian light sensitivity in the northern line (**a**, **b**) than in the southern line (**c**, **d**). The sigmoidal curve fits for sex and strain described the data significantly (F_10,26_ = 4.82, *p* < 0.0001)

To provide a better quantitative comparison of circadian light sensitivity for the different strains, we integrated the PRCs between ZT0 and ZT16 to provide a single phase shifting capacity value for each PRC. To compare the response capacity value to a single currency value for each light stimulus, we calculated the total photon dose provided by integrating light intensity over the duration of the stimulus. This data set now allows us to plot integrated circadian phase response against integrated stimulus strength for males and females of the different strains (Fig. 4).

**Fig. 5:**
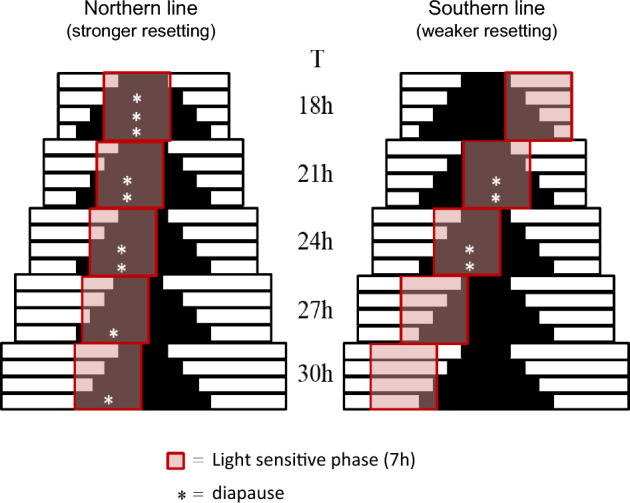
Graphical external coincidence – phase angel of entrainment (EX-PA) model explaining latitudinal variation in diapause induction using differences in light sensitivity. Higher light sensitivity (and thus stronger resetting) in the northern line results in a narrower distribution of phase angle of entrainment over the different T-cycles and photoperiods. This results in a narrower phase distribution of the light-sensitive phase (red box) over the T-cycles and as a result diapause occurs in all T-cycles (white asterisk). The southern line shows a wider phase angel of entrainment due to its lower light sensitivity and a wider distribution of the light-sensitive phase across the T-cycles. Consequently, diapause occurs in just two T-cycles with a period close to the intrinsic circadian period around 24 h

These results indicate that *Nasonia* females are less light sensitive than the males, but have a higher circadian response capacity than males (Fig. 4a, c vs. b, d). For both males and females, we see that northern line is more light sensitive than the southern line with about one order of magnitude differences (Fig. 4a, b vs. c, d; F_6,30_ = 10.64, *p* < 0.001). General oscillator theory would predict that for the northern strain this will result in less variation in phase angle of entrainment under different LD dark schedules. The consequences of this finding for the photoperiod responses in our partial Nanda-Hamner protocol will be further evaluated in the discussion (Fig. 5).

## Discussion

Our data compare the photoperiodic response of two *Nasonia* strains under extended T-cycles. Our experiments were not designed to confirm a positive Nanda-Hamner response showing circadian involvement in photoperiodism since this was already performed extensively in *Nasonia vitripennis* by Saunders using a full Nanda-Hamner landscape (Saunders 1968, 1974). Nonetheless, where there is overlap in treatment, our data are in line with those obtained by Saunders (Fig. 4). In addition, our comparison between the northern and southern line provides additional evidence that latitudinal adaptation in *Nasonia* may be caused by differences in properties of the circadian system, resulting in adaptive differences in photoperiodic responses. The northern *Nasonia* line shows in different T-cycles a positive response and variation in CPP; with longer CPP in longer T-cycles and shorter CPP in short T-cycles. These differences became slightly smaller when plotting the data as a function of critical night length, but still the curves did not superimpose. This may indicate that the photoperiodic system in the northern *Nasonia* line responds to both day length and night length.

Saunders collected large data sets when he applied night interruption experiments (Saunders 1970) and the Nanda-Hamner protocol (Saunders 1974). Both experimental approaches led to the conclusion of a circadian-based seasonal timing mechanism. Further, Saunders wanted to determine oscillator properties, especially whether internal or external coincidence model drives seasonal timing. At the time, he interpreted his findings as internal coincidence model based on the position of the ascending and descending slope of the peak of the photoperiodic response curve. *“…When the photoperiod was increased from 12 h to 14 or 16 h the ‘descending slope ‘ of the peaks remained in the same position (at T* = *28–32 and at T* = *52–56), but the ‘ascending slope’ moved to longer T values in such a way that the maxima became narrower. …. A similar, but opposite trend was observed when the photoperiod was shortened; the ‘ascending slope’ moved steadily to the left, whereas the ‘descending slope’ remained constant, except with the 4 h photoperiod in which a perceptible movement to left was also apparent.“* (Saunders 1974, see also Fig. 3). He connected the ascending slopes with dusk and the descending slopes with dawn and by that he drew the conclusion of an internal coincidence model with a morning oscillator tracking dawn and an evening oscillator tracking dusk (Saunders 1974). However, compelling and intuitive this interpretation is, it is important to realize that the photoperiod vs. T-cycle contour plots in Saunders 1974 (Figs. 1,  2) do not depict circadian phase of entrainment and cannot be used to say anything about phase relationships with ‘dawn’ or ‘dusk’. Considering the dynamic process of circadian entrainment, better distinction between internal and external coincidence timing models can only be obtained by dynamic simulation of the entrainment process under these various T-cycles.

Based upon our own data, we propose an alternative explanation based upon the more parsimonious external coincidence timing model, similar to the photoperiodic timing mechanism located in the mammalian *Pars tuberalis* (Dardente et al. 2010; Masumoto et al. 2010). Essential for evaluation of the involvement of circadian components in photoperiodic timing in unusual T-cycles is whether the circadian system is able to entrain or not, especially in absence of photoperiodic response as shown in the southern *Nasonia* line (Fig. 2c, d). Phase response curves from females of the northern and southern lines to light pulses of the same light intensity as used here, showed that light pulses of 4 h or more resulted in phase shifts of more than 7 h advance or delay (Table 1, Fig. s1). This means that both the northern (tau = 26.33 h) and the southern line (tau = 25.01 h) probably entrained to all T-cycles presented here. Assuming that circadian entrainment indeed occurred in both lines under all T-cycles, we consider the difference in light sensitivity of the northern and southern line (Fig. 4), as a possible explanation for the difference in diapause response. The higher light sensitivity in the northern line should result in a larger range of entrainment and therefore a less steep phase-period relationship under a range of T-cycles than would be the case with the southern line with a lower light sensitivity (Floessner and Hut 2017). This means that with increasing duration of the T-cycle, the southern line would show a stronger effect on phase (more ‘leading’) than the northern line. This indeed seems to be the case when activity profiles for the northern and southern lines are considered for T-20 h, T-24 h and T-28 h LD cycles (Floessner et al. 2019). When this difference in phase-period relationship between the northern and southern line is applied to an external coincidence model, we can construct a graphical representation model that summarizes our results (Fig. 5). In this model, we assume that diapause is suppressed when a single light-sensitive phase of ~ 7 h duration would be exposed to light. This light-sensitive phase would be coupled to the circadian system and is entrained around the middle of the light phase (Floessner et al. 2019). This parsimonious model can explain all diapause responses presented here, when lower light sensitivity in the southern line (Fig. 4) results in a reduced range of entrainment and a wider phase angle of entrainment range (Floessner and Hut 2017) when compared to the northern line (Fig. 5). The narrower range of phase angle of entrainment in the northern line, resulting from their higher light sensitivity, would then lead to a narrower phase distribution of the light-sensitive phase, and hence more similar responses in maintaining diapause at shorter photoperiods at all T-cycles applied (Figs. 3, 5). The wider phase distribution in the southern line, due to their lower circadian light sensitivity, would therefore result in maintenance of diapause only in those T-cycles with a period relatively close to the intrinsic circadian period of that line.

Our ‘external coincidence—phase angle of entrainment’ (EX-PA) model seems in line with the interpretation by Vaze and Helfrich-Förster (2016) that a tight range of phase angle of entrainment may indicate either a weak or dampened oscillator that can be easily entrained by light, or a strong oscillator that can be easily entrained because of a high response/sensitivity to light. Under entrained conditions, these two options can only be distinguished by an independent measure of oscillator strength, perhaps through establishing the robustness of activity rhythms, and by measuring the strength of the circadian light responses. Both measures, robustness of activity profiles and strength of circadian light resetting, seem to indicate that *Nasonia* has a strong circadian oscillator with strong circadian light resetting, especially in the northern line (Tables 1, 2; Fig. 4). As a next step, it would be interesting to see to what extent our EX-PA model can explain the full extent of the photoperiodic landscape model as measured by Saunders (1974).

To further support our EX-PA model, we would need better confirmation on the phase angle of entrainment under various T-cycles and we would need to increase our understanding of the system using a wider range of photoperiods and T-cycles applied to a wider range of *Nasonia* strains. Given the results presented here, we draw the preliminary conclusion that the different diapause responses in our northern and southern lines can be explained (at least partly) by an external coincidence timing model while taking into account the higher circadian light sensitivity in the northern line (Fig. 4). This interpretation indicates that variation in circadian light sensitivity, together with adaptation in circadian period, may form the basis of adaptation to geographically variable seasonality by photoperiodic timing of diapause. However, our interpretation should be taken with caution since it is merely based on 2 isofemale lines. It, therefore, remains to be tested in a wider range of lines per location and over more locations before firm conclusions can be drawn.

In a follow-up study, Vaze et al. (2023, this issue) concluded that the bimodal activity patterns in *D. ezoana* seem to be driven by an evening and, perhaps to a lesser extent, a morning oscillator. However, when testing for diapause induction they concluded that *D. ezoana* explicitly measures night length, which is in line with a single dampened oscillator driving diapause, thus an external coincidence timing model (Vaze and Helfrich-Förster 2016). This indicates that different circadian oscillators may regulate the different behaviours: activity and diapause. It is important to recognise that our data *can* be explained by an external coincidence timing model, but they do not exclude the possible existence of more complex internal coincidence timing that requires two oscillators (Saunders 2023, this issue). In fact, the observation that *Nasonia* measures a mixture of both day length and night length can be viewed as evidence that an internal coincidence timing model with two oscillators is at play. However, this type of evidence is indirect at best, and neglects the complexity of the dynamical system that entrainment of a single circadian oscillator is. Such dynamical systems are typically described by their non-linear nature, leading to non-intuitive outcomes for driving diapause. It is good to recognize that it is only when obtain direct measurements of the circadian oscillators involved, that we can decisively distinguish between internal and external coincidence timing models to explain diapause induction.

Our finding of increased circadian light sensitivity in the northern line seems at variance with the interpretation of Pittendrigh and Takamura (1989) based on *Drosophila auraria* in Japan. In their paper they deduced PRC amplitude from differences in phase angle of entrainment in eclosion rhythms, indicating lower PRC amplitude towards the north. Their functional interpretation was that lower light sensitivity could (1) maintain entrainment at high latitudes when night length becomes critically short, and that (2) decrease in pacemaker amplitude at extremely long photoperiods could be mitigated by reducing circadian light sensitivity. It is important to note that both interpretations were based upon modelling observations using the Pavlidis oscillator model and not on actual measurements of amplitude. Indeed, they indicated themselves that a change in circadian light sensitivity would also have consequences on the phase angle of entrainment of the circadian system, which is a feature that we explored here. Pittendrigh and Takamura (1989) further indicate the importance of describing a latitudinal cline in circadian light sensitivity, but they realized that *“Needless to say, if such a rule is found, there will be plenty of exceptions: Like that proverbial skinner of cats, natural selection tends to find many ways of doing its job.”* Even though our study is limited by the lack of genetic variation, our data do emphasize that circadian light sensitivity may play a crucial role in local adaptation of the photoperiodic response, albeit opposite to the earlier prediction of Pittendrigh and Takamura (1989).

### Supplementary Information

Below is the link to the electronic supplementary material.

Supplementary file1 (DOCX 785 KB)

## Data Availability

Data can be made available by the authors upon request.
