# Supplementary material for: Variation in photoperiod response corresponds to differences in circadian light sensitivity in northern and southern Nasonia vitripennis lines
Source: J Comp Physiol A Neuroethol Sens Neural Behav Physiol. 2023 Oct 18;210(4):667–76. doi: 10.1007/s00359-023-01674-2 (PMC11226509; doi:10.1007/s00359-023-01674-2)
Supplement: Supplementary file 1 — Supplementary file1 (DOCX 785 KB) [file 359_2023_1674_MOESM1_ESM.docx]

Variation in photoperiod response corresponds to differences in circadian light sensitivity in northern and southern *Nasonia vitripennis* lines.

Theresa S.E. Floessner^1^, Elena Dalla Benetta^2^, Domien G.M. Beersma^1^ and Roelof A. Hut^1*^

^1^ Chronobiology unit, Neurobiology expertise group, Groningen Institute for Evolutionary Life Sciences, University of Groningen, the Netherlands.

^2^ Evolutionary Genetics, Development & Behaviour expertise group, Groningen Institute for Evolutionary Life Sciences, University of Groningen, the Netherlands.

* Correspondence: [r.a.hut@rug.nl](mailto:r.a.hut@rug.nl)

**Materials & Methods**

*Experimental lines and maintenance*

The experiments were performed with the parasitoid wasp *Nasonia vitripennis*; with lines originating from Oulu, Finland (65.01°N) and Corsica, France (42.04°N) (Paolucci et al. 2013). Most experiments were conducted with one isofemale line from Oulu (northern line) and one isofemale line from Corsica (southern line) established by Paolucci et al. 2013. Other lines from the Oulu and Corsica region were used for control experiments. All lines were reared in a temperature and humidity controlled climate chamber (20 ±1°C, 50‑55% RH) in a light-dark cycle of 16-h of light (646 lux) and 8‑h of darkness per day (LD 16:8) to prevent diapause induction. All individuals were offspring from separately housed females that were presented to *Calliphora spp.* pupae as hosts.

*Locomotor activity measurements*

Circadian phase shifts to single light pulses at different times of day were conducted in an Aschoff type II experiment (Aschoff 1965). All animals, independent from the time of light pulse, receive the same light-dark cycle (LD) of 16h:8h during the first five consecutive days, followed by constant darkness (DD) where the light pulse was given after two days of DD; different groups of individuals received group specific single light pulses at 12 different time points in 2-h intervals. As circadian output we recorded locomotor activity of individuals by using the Drosophila Activity Monitoring System (DAMS, by TriKinetics, Waltham, USA). To detect the new phase after the light stimulation the recording continued for at least seven consecutive days in constant darkness and compared to individuals which did not receive any light pulses. For the activity recording, individuals (eight per experimental group and condition) of the age of three to five days, fertilized females and males, were transferred into activity tubes (6.5 x 0.5 cm), filled one quarter with agar food (30% sucrose, 1.5% agar, 0.15% nipagin) and closed with a cotton plug on the other end. The tubes fit into recording monitors (32 tubes in one monitor) that recorded activity per minute of each individual separately by infrared light beams that were interrupted when a wasp crossed it. The monitors were placed into light-tight boxes (23 x 14 x 32 cm), in 18°C (±1 °C) and 50-­55% RH. Each light-tight box was illuminated with one LED light source (Neutral White 4000K, PowerStar, Berkshire) of providing maximally 2.10 · 10^15^ photons·cm^-2^·s^-1^ (high light intensity). To decrease light intensity we inserted neutral density filters into the light-tight boxes two hours before “light off” of the last LD cycle. Filters reduced light intensity to 2.62 · 10^14^ photons·cm^-2^·s^-1^ (intermediate light intensity) and 9.37 · 10^13^ photons·cm^-2^·s^-1^ (low light intensity).

*Determination of Phase Shifts*

Phase shifts were determined in ChronoShop (Spoelstra et al. 2004) individually by comparing old phase before the light pulse, and new phase, after the light pulse (excluding the first two transient days after the light pulse). As a phase marker for the activity rhythm we used centre of gravity (Kenagy 1980). In average 12% of individuals were excluded when the visual inspection of the actograms showed arrhythmicity (usually Lomb-Scargle dPn value<15) or abnormal activity patterns. The timing of the light pulse was calculated relative to the light dark cycle and the phase shift was calculated to a dark control for each sex and strain specifically. Because phase shifts may exceed 12 h, we used circular averages by calculating the average vector on a circular 24-h scale. Phase response curves were plotted as average phase shift (h) against time of mid pulse (ZT, h). Classification into strong (type 0) or weak (type 1) phase resetting was obtained by visual inspection by triple plotting the abscissa and ordinate (both being circular time scales) of the phase transition plots (new phase vs. old phase). This yields clear horizontal patterning (slope=0) of the data in the case of type 0 phase transition curves (PTCs) and slant patterning (slope=1) in the case of type 1 PTCs. We obtained clear horizontal (type 0) or slanted (type 1) patterns, allowing for clear determination of strong or weak resetting for all obtained phase shift curves.

Light pulses of various duration (0.3, 1, 4, 8, 16 h) and intensity (9.37*10^13^ (low); 2.62 · 10^14^ (intermediate); 2.10 · 10^15^  (high) photons·cm^-2^·s^-1^) were applied. By multiplying duration and intensity, we calculated photon dose (in photons·cm^-2^) for each combination of intensity and duration. To calculate photon dose response curves within each line and sex, each PRC was collapsed into a single value by integration towards the 0h phase shift axis between ZT0 and ZT16. This integral coincides with the light phase during previous entrainment and allowed for selective evaluation of entraining circadian light responses. Although there is no clear distinction between large advances and large delays in type 0 phase response curves, our method of restricted integration allowed us to avoid the discontinuous transition between advances and delays which would otherwise prevent unambiguous calculation of the integral. Photon dose response curves through these integral values were fitted using a modified Naka-Rushton equation (Hut et al. 2008).

**Fig. s1.** Phase response curves (PRCs) from individually recorded Nasonia vitripennis females from northern and southern Europe. Light pulses of different durations (indicated on the left side of each graph-pair) and high light intensity were applied. Circadian phase shifts (h) are plotted against time of mid-light pulse (ZT, h). Open circles represent individual phase shifts, connected solid circles represent circular averages. Shorter light pulse durations caused smaller phase shifts (type 1 PRC) whereas longer light pulses have led to bigger phase shifts (type 0 PRC). A transition from weak (type 1) to strong (type 0) PRCs occurred in the northern line with light pulses of 4 h or longer and in the southern line with light pulses of 8 h or longer.

**Fig. s2.** Phase response curves (PRCs) from individually recorded Nasonia vitripennis males from northern and southern Europe. Light pulses of different durations (indicated on the left side of each graph-pair) and high light intensity were applied. Circadian phase shifts (h) are plotted against time of mid-light pulse (ZT, h). Open circles represent individual phase shifts, connected solid circles represent circular averages. Shorter light pulse durations caused smaller phase shifts (type 1 PRC) whereas longer light pulses have led to bigger phase shifts (type 0 PRC). A transition from type 1 to type 0 PRCs occurred in northern line with light pulses longer than 1 h and in the southern line with light pulses longer than 4 h.

******

**Fig. s3**. PRCs using 1-h light pulses at three different intensities from individually recorded Nasonia vitripennis females from northern and southern Europe. Low, intermediate and high light intensity pulses (indicated on the left side of each graph-pair). Circadian phase shifts (h) are plotted against time of mid-light pulse (ZT, h). Open circles represent individual phase shifts, solid circles and lines represent circular averages. Both lines show weak resetting (type 1 PRCs), while in general the northern line expresses bigger phase shifts than the southern line in all three light conditions.

**Fig. s4.** PRCs using 1-h light pulses at three different intensities from individually recorded Nasonia vitripennis males from northern and southern Europe. Low, intermediate and high light intensity pulses (indicated on the left side of each graph-pair). Circadian phase shifts (h) are plotted against time of mid-light pulse (ZT, h). Open circles represent individual phase shifts, solid circles and lines represent circular averages. In all three light intensities the northern line shows strong resetting (type 0 PRCs) and the southern line weak resetting (type 1 PRCs). Within the lines there are no big differences between the different light conditions.

**Fig. s5.** PRCs using 4-h light pulses at three different intensities from individually recorded Nasonia vitripennis females from northern and southern Europe. Low, intermediate and high light intensity pulses (indicated on the left side of each graph-pair). Circadian phase shifts (h) are plotted against time of mid-light pulse (ZT, h). Open circles represent individual phase shifts, solid circles and lines represent circular averages. The northern line responds with strong resetting, type 0 PRCs, to all three intensities, whereas the southern shows type 1 PRCs to all three intensities.

**Fig. s6.** PRCs using 4-h light pulses at three different intensities from individually recorded Nasonia vitripennis males from northern and southern Europe. Low, intermediate and high light intensity pulses (indicated on the left side of each graph-pair). Circadian phase shifts (h) are plotted against time of mid-light pulse (ZT, h). Open circles represent individual phase shifts, solid circles and lines represent circular averages. Both lines showed strong resetting (type 0 PRC); the northern line showed even bigger phase shifts, especially the phase advances, than the southern line.
